# Effectiveness of a 2-dose varicella vaccination program in Changzhou, China, during the transitional period (2017–2022): a registry-based case-cohort study

**DOI:** 10.1186/s12889-025-21348-9

**Published:** 2025-01-17

**Authors:** Suting Xiong, Changlei Han, Dan Wu, Xiaomeng Mi, Peipei Zhang, Han Gao, Gan Cao, Fang Yao, Cong Chen, Xufeng Lv

**Affiliations:** 1 Changzhou Center for Disease Control and Prevention, No. 203 Taishan Road, Xinbei District, Changzhou City, Jiangsu Province 213000 China; 2 Changzhou Institute for Advanced Study of Public Health, Nanjing Medical University, No. 203 Taishan Road, Xinbei District, Changzhou City, Jiangsu Province 213000 China; 3 Changzhou Commission of Health, No. 1280 Longcheng Avenue, Xinbei District, Changzhou, Jiangsu Province China

**Keywords:** Varicella Vaccine (VarV), Vaccine Effectiveness (VE), Case Cohort study (CCS), Vaccination Program

## Abstract

**Background:**

The benefits of improving coverage and timeliness of varicella vaccination need to be quantified in countries where varicella vaccine (VarV) has not yet been included in national immunization programs. This longitudinal study analyzed the vaccine effectiveness (VE) of the varicella vaccination program implemented in Changzhou City during the transitional period (2017–2022).

**Methods:**

Using the Immunization Information System and National Notifiable Infectious Disease Surveillance System registry data, this retrospective case-cohort study assessed the VEs of varicella vaccination for Changzhou children born from 2016 to 2021. The subcohort was sampled using inverse probability weighting for the survival analysis design to evaluate the VEs under different dosages and cost types, eliminating confounding by age and location area. Sensitivity analysis for the exclusion part assessed the study's robustness.

**Results:**

A total of 5,172 children (1.12%) were sampled to the subcohort for studies, including 2,299 cases. VEs significantly rose with each successive dose, reaching 82.54% and 97.91%. VEs were lower for the single-dose subgroups until 2020, with significant increases in VEs in all subgroups in 2020 and after that. Most children who did not benefit from the “1–4” Vaccination Program had lower VEs due to delayed vaccination.

**Conclusion:**

Improving 2-dose VarV vaccine coverage and timely vaccination could enhance the immune barrier for susceptible children. During the transitional period, the “1–4” Vaccination Program of VarV positively impacted coverage and timeliness in Changzhou.

## Background

Varicella is a highly contagious respiratory disease caused by infection with the varicella-zoster virus (VZV). The secondary attack rate in susceptible populations has been documented to exceed 90% [1, 2]. The WHO recommends universal varicella vaccination with at least one dose at 12–18 months of age and 80% vaccine coverage in areas where varicella is a major public health issue [3, 4]. A variety of countries, including Australia, Germany, Japan, and the US, employ the varicella vaccine (VarV) to prevent and reduce varicella [2, 5–7]. The imported VarV was introduced to the Chinese market in 1997, followed by the successive authorization of domestic VarV production after 2000. From then on, people could voluntarily and at their own expense receive the single-dose VarV [8]. However, this vaccine has not yet been part of China’s National Immunization Program (NIP) [9].

China reported varicella as the third most common vaccine-preventable infectious illness after tuberculosis and influenza, and families and society are burdened [10, 11]. An official national survey found about 150 thousand varicella cases in Jiangsu Province, making it the province with the highest disease burden in 2019 [10]. China’s National Notifiable Infectious Disease Surveillance System (NNIDSS) estimated varicella incidence rising from 24/100,000 in 2010 to 70/100,000 in 2019 [10]. Beijing [12], Shanghai [13], Hangzhou [14], Ningbo [15], Qingdao [16], and certain cities in Jiangsu Province [17–20] had successively adopted 2-dose VarV policy support into local vaccination programs between 2012 and 2020. However, the vaccination coverage of these programs varies widely by region [21–25]. A comprehensive meta-analysis assessing varicella vaccination coverage among Chinese children revealed significant disparities across provinces, with the pooled coverage reaching 97.3% in the eastern region compared to 40.8% in the central and western regions [26]. Additionally, another meta-analysis study highlighted the variability in vaccination rates among school-age children in China, with VarV_1_ coverage ranging from 48.80% to 98.41% and VarV_2_ coverage ranging from 23.46% to 88.67% [27].

The incidence of varicella in Changzhou, Jiangsu, also increased significantly from 2011 to 2019. During the period, the single-dose VarV vaccination coverage for children aged 1–6 years in this city was around 58%, significantly below the WHO-recommended ≥ 80% [5]. Based on the high incidence and burden of varicella, Changzhou has constructed a strategy for VarV vaccination called the “1–4” Vaccination Program. Beginning on January 1, 2020, Changzhou children born on or after January 1, 2019, who are 1 year old and have not had varicella, will receive two free VarV doses: one at 1 year and one at 4 years. Non-infected children born after 2016 but under 4 received one free VarV dose. People who meet the conditions can also pay for other VarVs. An Immunization Information System (IIS) ensures that each age-appropriate child can be vaccinated only twice, regardless of whether they choose the free or the paid vaccine option.

In Changzhou, the population-wide incidence rate of varicella had soared to its highest point in 2019, reaching 259.63 cases per 100,000 individuals, following the classification of varicella as a “Class C” notifiable infectious disease in Jiangsu Province in 2017 [28]. From 2016 to 2019, the vaccination coverage for a single dose of VarV among children aged 1–6 years in Changzhou was around 50%, which was lower than the WHO’s recommended rate of ≥ 80%. Subsequently, by 2022, Changzhou had a 1-dose VarV vaccination rate of > 95% among children aged 1 to 6 years, and population-wide varicella incidence dropped to 63.58/100,000 [28]. Nevertheless, it is insufficient to evaluate the effect of the new strategy just on vaccination coverage and disease incidence alone. An authoritative review study has demonstrated that non-pharmaceutical interventions during the COVID-19 pandemic significantly reduced the incidence of respiratory infectious diseases [29]. Vaccine effectiveness (VE) assessment is an epidemiological study that measures the proportion of cases in which certain vaccines prevent and avert disease in real life.

The Case Cohort Study (CCS) was conducted to estimate VE in immune subgroups by dose and cost type [22, 30]. This method picks a sample similar to a case–control study from a prospective cohort to calculate hazard ratio (HR) without collecting complete data for the total cohort [31]. The subcohort consists of controls from the total cohort and those who develop cases during follow-up. This study strategy reduces study costs, reduces sample size, and maintains robust causal inference [32].

This study was situated within a pivotal transitional phase in the varicella vaccination program of Changzhou, China, from 2017 to 2022. This era was characterized by a series of transformative policy shifts. In 2017, within the framework of a nationwide policy in China that advocated voluntary, self-funded single-dose varicella vaccination, Jiangsu Province mandated varicella surveillance as a “C” infectious disease, encompassing the city of Changzhou [33]. A significant milestone was reached in 2020 when Changzhou embarked on a free two-dose vaccination program, a precursor to the broader provincial implementation of a similar policy in 2023. This transitional period (2017–2022), thus, represented a dynamic interval during which the effectiveness of varicella vaccination was evaluated across varying dosages and cost types. Our investigation evaluated the effects of one and two doses of the VarVs. Also, it delved into the influence of vaccine cost policies on immunization coverage and disease incidence, providing insights into the evolving immunization programs during this critical period. More high-quality, longitudinal epidemiologic evidence is needed to adopt VarVs in the NIP.

## Methods

### Data source

We conducted the CCS using VarV data from the IIS [34] and varicella diagnosis data from the NNIDSS. The IIS in Jiangsu Province meticulously records immunization data for children aged 0–6 years. This comprehensive database includes detailed vaccination information, such as administered doses and cost types, and essential demographic details, like date of birth, gender, and residential location. The NNIDSS reports infectious diseases nationwide. It describes case diagnosis and demographics.

Given that multiple studies have demonstrated the impact of place of residence and age on the sensitivity of varicella reporting [35, 36], this study was meticulously designed to address the potential discrepancies in surveillance sensitivity. By employing stratified sampling based on the joint distribution of residential location area and age of the case group, we aimed to mitigate these differences. Specifically, we divided the population into 42 distinct strata, derived from the 7 administrative areas of Changzhou City and the six age groups ranging from 1 to 6 years. People could refer to the well-established stratified sampling methodologies, as elegantly described by Borgan et al. (2000) [37].

### Identification of varicella cases

All patients aged 1–6 years newly diagnosed with varicella and reported to the NNIDSS from January 1, 2017, to December 31, 2022, were identified, including: 1) Clinically diagnosed cases: a typical clinical presentation with a history of exposure to varicella or herpes zoster. 2) Confirmed cases: require laboratory evidence, including nucleic acid or serum IgM antibody. Cases without immunization profiles in the IIS were excluded using unique identifiers.

### Validity of immunization

For those born from January 1, 2016, to December 31, 2021, the VarV records of this birth cohort administered by the IIS were identified: 1) “1–4” Vaccination Program: Valid 1-dose VarV should be administered at and after 12 months and before 4 years, and valid 2-dose VarV should be administered at and after 4 years and before 6 years. 2) Pre-diagnostic vaccination: Valid vaccinations of cases should be earlier than 14 days prior to reporting by the NNIDSS. Individuals who did not follow the “1–4” Vaccination Program were excluded, and dosages that did not meet the pre-diagnostic vaccination criteria were considered invalid.

### Definition of other variables

The variable “Type” within the cohort was designated to categorize the vaccination status based on the funding source of the VarV doses. The categories were defined as follows: “N” for zero-dose (no vaccination), “F” for free-dose (vaccination provided free of charge), and “O” for self-pay-dose (vaccination paid for by the individual). This classification resulted in “X–Y” combinations that represented the vaccination status for the first and second doses, respectively, including: “N–N” (no vaccination for both doses), “O-N” (self-pay for the first dose, no second dose), “F-N” (free for the first dose, no second dose), “O-F” (self-pay for the first dose, free for the second dose), “F-O” (free for the first dose, self-pay for the second dose), “O–O” (self-pay for both doses), and “F-F” (free for both doses). These combinations provided a comprehensive overview of the vaccination patterns within the cohort, facilitating a detailed analysis of the impact of cost types on vaccine coverage and effectiveness.

The “Reason” variable was a critical indicator in the exclusion dataset, designed to elucidate the rationale behind the exclusion of certain individuals from the primary vaccine effectiveness study based on case-cohort analysis. This dataset retained the vaccination-disease onset sequence but lacked a definable time variable due to non-compliance with the “1–4” Vaccination Program, necessitating another logistic regression analysis.

The “Reason” variable categorized individuals into four labels based on their vaccination status relative to the “1–4” Program: “None” (no vaccination), “Early” (vaccination before the recommended age), “Timely” (vaccination following the “1–4” Program), “Late” (vaccination after the recommended age), and “Invalid” (vaccination not following Pre-diagnostic vaccination). This classification resulted in “X–Y” combinations that represented the vaccination status for the first and second doses, respectively, including: “L-N” (Late-None), “E-N” (Early-None), “L-I” (Late-Invalid), “T-I” (Timely- Invalid), “L-L” (Late-Late), “L–T” (Late-Timely), “T-L” (Timely-Late), “T-E” (Timely-Early), “E-L” (Early-Late), “E-T” (Early-Timely), and “E-E” (Early-Early).

### Sample rate of the controls in subcohort

Using the formula (1), the subcohort sampling procedure in the case-cohort design derived *q*(%) as 1.12% [38]. The study parameters included: *p*_D_ = 877.73 per 100,000 children, 2-dose vaccination coverage *p*_1_ = 28.29%, *p*_2_ = 1- *p*_1_, vaccine effectiveness (1-θ) of 80% in the literature, *α* = 0.05, and *β* = 80%.

1 $$q\left(\%\right)=N\times B\times p_D/\left(N-B\times\left(1-p_D\right)\right),\;where\;B=\left(Z_{1-a}+Z_\beta\right)^2\;/\;\theta^2\times p_1\times p_2\times p_D$$

### Incidence rate calculation

The incidence rate was determined by dividing the number of new cases among children aged 1–6 years, born in Changzhou between 2016 and 2021, who developed the disease between 2017 and 2022 by the total number of children in the same age group and birth cohort. The rate is expressed per 100,000 children by multiplying the result by 100,000.

### Vaccine effectiveness

Cox proportional hazards models can calculate HR values and its 95% CI using the outcome event (varicella or not) and observation time as dependent variables, thereby yielding the VE [39]. By vaccine dose, we identified subcohort cases and controls’ observation time. In this study, a child who received a given number of vaccine doses was at risk of developing varicella during the observation time, and we assumed constant VZV transmission [30].

The maximum cumulative observation time for the control child was 59.5 months, ranging from 12.5 months to the child’s 6th birthday. This time period was between the age of 12.5 months and the onset of illness in the cases. The total observation time for all patients and controls was divided into three subgroups based on the number of VarV doses received: “0-dose”, “1-dose”, and “2-dose”. The HRs obtained by case-cohort survival analysis were used to calculate the VEs using the formula (2) [30]:

2 $$\mathrm{Vaccine}\;\mathrm{effectiveness}\;(\mathrm{VE})=\left(1-\mathrm{HR}\right)\times100\%$$

### Statistical analysis

Categorical baseline characteristics such as gender, age, and location area were compared using the chi-squared test, and the continuously skewed variable median observation time was analyzed using the rank-sum test. R 4.3.3 was used for statistical analysis and figure drawing, with significance determined using two-tailed tests with *α* = 0.05.

The Kaplan–Meier curve was used to analyze the effect of a single factor on the VEs during the observation period. Using inverse probability weighting (IPW), a Cox proportional hazards model calculated VEs (the R package “*cchs*”) for each subgroup of vaccine doses, eliminating confounders and sampling probabilities. The VEs were quantified in the Nomogram, which quantifies individual score lines for each item and generates a cumulative score line, the Total Score, which predicts the probability of incidence over the next 1–5 years. Higher scores on the Nomogram indicate a higher risk of incidence.

### Sensitivity analysis

To demonstrate timeliness’s effect, logistic regression and a heat map were applied within an exclusion dataset. Sensitivity analysis examined the impacts of dose levels, types of costs, and exclusion reasons using three logistic regression models, adjusted for age and sex. Logarithmic transformation normalizes graph data to increase heat map resolution.

## Results

### Study population included

The IIS system collected 337,311 immunization records for Changzhou, Jiangsu’s 2016–2021 birth cohort aged 1–6 years by December 31, 2022. A total of 268,691 (79.66%) met the Changzhou “1–4” Vaccination Program.

Between 2017 and 2022, varicella incidences for children aged 1–6 varied from 179.05 (in 2022) to 1,951.19 (in 2019) per 100,000. In this study, the NNIDSS reported 2,512 cases from the birth cohort of laboratory- or clinical-confirmed varicella. 2,299 (91.52%) met the study’s case-inclusion criteria by screening the IIS records.

**Fig. 1:**
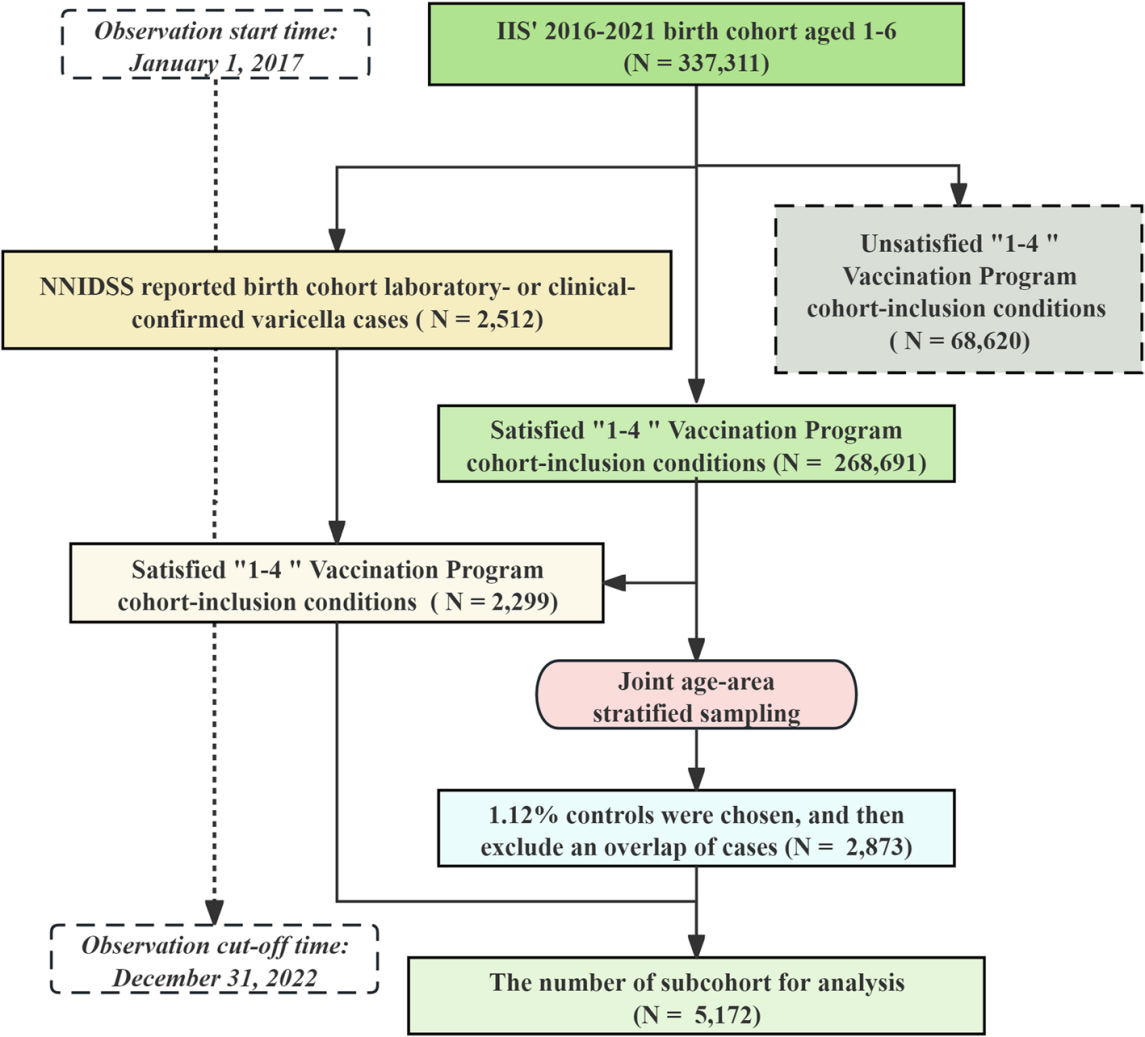
Flowchart of including and excluding individuals for estimating VE in Changzhou, Jiangsu 2017–2022

2,873 individuals were selected from the cohort using the subcohort sampling method, with a sampling ratio of 1.12%. The sample size of the subcohort, which included each case without any overlap, was 5,172. Figure 1 illustrates a clear pattern for subcohort selection.

### Characteristics of the case, cohort, and subcohort populations

The control subjects of subcohort (Group 2) did not have statistically significant differences in the distribution of variables of demographic characteristics from the cases (Group 3), which differed from the characteristics displayed by controls in the total cohort (Group 1). Cumulative VarV coverages in Group 2 were almost 74.45% for the first dose, 55.45% for the second, and 25.55% without VarV. Quite differently, 14.62%, 2.22%, and 85.38% of Group 3 received 1, 2, and 0 doses of the VarV vaccination, respectively. In the subgroups by the cost type, the top three types for Group 2 were: “O-F”, “N–N”, and “O-N”. Group 3 consisted of 85.38% “N–N” type, with the remaining percentages distributed as follows: “O-N”, “F-N”, and “O-F”. Group 3 had a median observation time of 824 days (IQR: 438–1,011 days) for cases, while Group 2 had 1,701 days (IQR:1,242–1,825 days). The relevant information is shown in Table 1.

**Table 1:** Characteristics of the case, cohort control, and subcohort control populations

Variables	Group 1 Control subjects of Total cohort	Group 2 Control subjects of Subcohort	Group 3 Case group	*P*_*T*_	*P*_*S*_
N	266,392	2,873	2,299		
Gender (%)					
Female	127,823 (47.98)	1,375 (47.86)	1,103 (47.98)	1.000	0.955
Male	138,569 (52.02)	1,498 (52.14)	1,196 (52.02)		
Dose (cumulative %)				< 0.05	< 0.05
0	48,130 (18.07)	734 (25.55)	1,963 (85.38)		
1	218,262 (81.93)	2,139 (74.45)	336 (14.62)		
2	74,041 (27.79)	1,592 (55.41)	51 (2.22)		
Type (%)				< 0.05	< 0.05
N–N	48,130 (18.07)	734 (25.55)	1,963 (85.38)		
O-N	26,676 (10.01)	359 (12.50)	181 (7.87)		
F-N	117,545 (44.12)	188 (6.54)	104 (4.52)		
F-F	6,243 (2.34)	85 (2.96)	0 (0.00)		
F-O	720 (0.27)	10 (0.35)	0 (0.00)		
O-F	65,480 (24.58)	1,455 (50.64)	51 (2.22)		
O–O	1,598 (0.60)	42 (1.46)	0 (0.00)		
Age(%)				< 0.05	0.999
1	37,223 (13.97)	12 (0.42)	10 (0.43)		
2	43,520 (16.34)	36 (1.25)	28 (1.22)		
3	54,929 (20.62)	130 (4.52)	103 (4.48)		
4	45,853 (17.21)	461 (16.05)	362 (15.75)		
5	42,423 (15.93)	896 (31.19)	712 (30.97)		
6	42,444 (15.93)	1,338 (46.57)	1,084 (47.15)		
Area (%)				< 0.05	1.000
LiYang County	36,258 (13.61)	465 (16.19)	378 (16.44)		
JinTan District	24,531 (9.21)	159 (5.53)	125 (5.44)		
Wujin District	70,435 (26.44)	763 (26.56)	608 (26.45)		
Xinbei District	51,308 (19.26)	538 (18.73)	430 (18.70)		
Tianning District	27,507 (10.33)	389 (13.54)	313 (13.61)		
Zhonglou District	32,460 (12.19)	405 (14.10)	324 (14.09)		
JinKai District	23,893 (8.97)	154 (5.36)	121 (5.26)		
Median observation time (days)[IQR]	1,065.00 (603.00, 1,509.00)	1,701.00 (1,242.00, 1,825.00)	824.00 (438.00, 1,011.00)	< 0.05	< 0.05

Table 1 presents cross-sectional information as of December 31, 2022

Dose: varicella vaccine dosage; Type: cost types for varicella vaccine (Note: N = None, O = One dose, F = Self-funded. For example, “O-N” indicates One dose self-funded, no subsequent vaccination. Other combinations are to be interpreted similarly.); Area: chhildren's residence in Changzhou districts and county; Median observation time: time of observation when 50% of individuals remain free of varicella; P_T_: *P*-value for case group vs. Total cohort; P_S_: *P*-value for case group vs. Subcohort

### Survival curves and median observation times

**Fig. 2:**
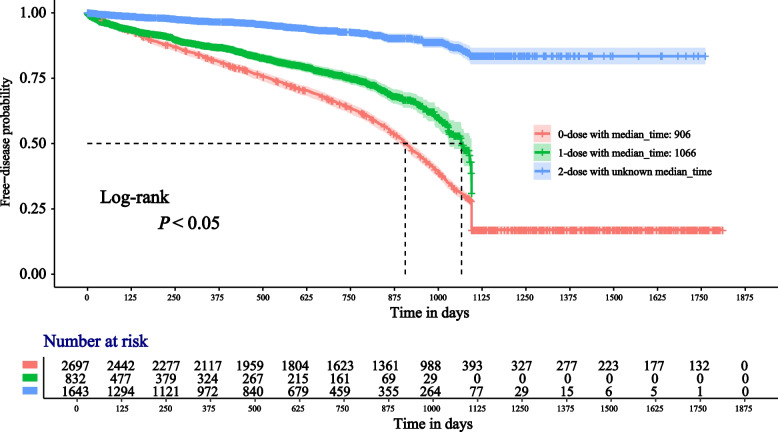
Survival curves by dose. (Median observation times were labeled in the legend; Fig. 2 was divided into “0-dose”, “1-dose”, and “2-dose” subgroups)

**Fig. 3:**
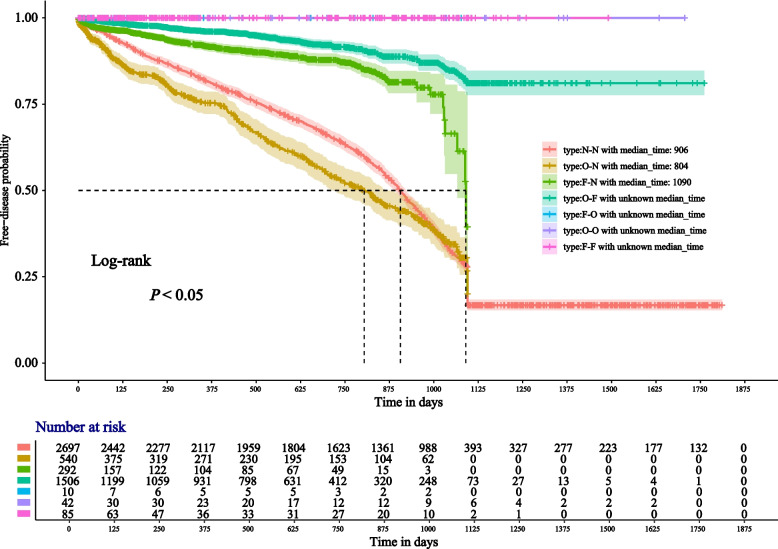
Survival curves by cost. (Median observation times were labeled in the legend; Fig. 3 was divided into seven subgroups: “N–N”, “O-N”, “F-N”, “O-F”, “F-O”, “O–O”, and “F-F”; Note: N = None, O = One dose, F = Self-funded. For example, “O-N” indicates One dose self-funded, no subsequent vaccination. Other combinations are to be interpreted similarly.)

The 0-dose subgroup had a median observation time of 906 days and the 1-dose subgroup 1066 days. After two doses, almost 75% of people were healthy at the observation cutoff (December 31, 2022). The relevant information is shown in Fig. 2. Median observation times were 906 days for the “N–N” subgroup, 804 days for the “O-N” subgroup, and 1090 days for the “F-N” subgroup. The remaining subgroups’ median observation times were not achieved for the reasons above. The relevant information is shown in Fig. 3.

**Fig. 4:**
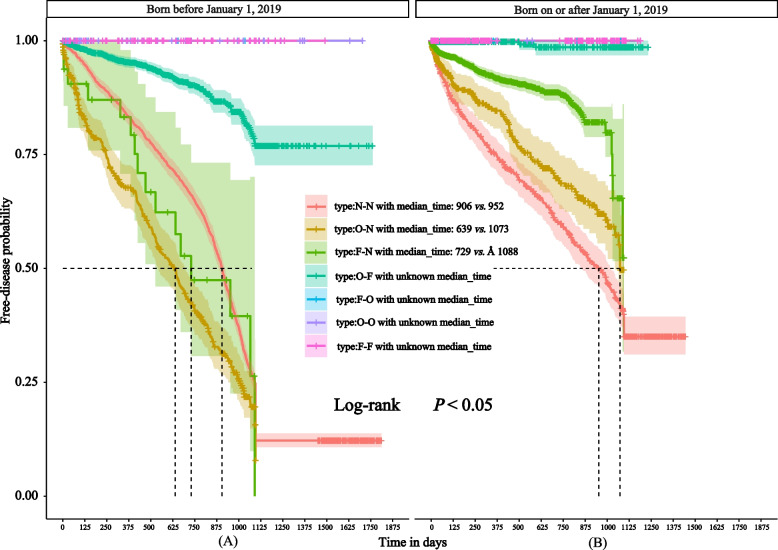
Two cost-type-related survival curves with January 1, 2019 as the cut-off. (Median observation times were labeled in the legend; children in Fig. 4A were born before January 1, 2019; Fig. 4B were born on and after January 1, 2019. Note: N = None, O = One dose, F = Self-funded. For example, “O-N” indicates One dose self-funded, no subsequent vaccination. Other combinations are to be interpreted similarly.)

In the “N–N” subgroup, “O-N” subgroup, and “F-N” subgroup shown in Fig. 4A (born before January 1, 2019), the median observation time for children over four years of age was 906 days, 639 days, and 729 days, respectively. The median observation times for children under four years of age in the “N–N” subgroup, the “O-N” subgroup, and the “F-N” subgroup shown in Fig. 4B (born on or after January 1, 2019) increased to 952, 1073, and > 1088 days, respectively.

Figure 3 indicates that the “O-N” curve is usually below the “N–N” curve and crosses over after 1000 days. The median “O-N” observation time is also 100 days less than the “N–N” kind. When we analyzed the subgroups using the age of four as the cutoff, among children born before January 1, 2019 (Fig. 4A), the “N–N” curve was above the “O-N” and the “F-N” curves, even if the confidence interval for the latter was too big. When we analyzed the subgroups using January 1, 2019, as the cutoff, the ordering of the curves in Figs. 4A and 4B closely corresponds to the vaccine effectiveness (VE) estimates derived from the Cox proportional hazards models, as discussed in detail in the Comparison differences in VEs between subgroups section.

### Comparison differences in VEs between subgroups

In Model 1, VEs (95% CI) against varicella increased with each dose, reaching 82.54% (78.88%, 85.57%) and 97.91% (96.91%, 98.59%). Those who received two VarV doses had higher VEs against varicella in the subgroups “O-F”, “F-F”, “O–O”, and “F-F” (more than 95%). In subgroups “O-N” and “F-N” who received only one VarV, VEs were weaker. Note that subgroup “O-N” has 18% lower VE than “F-N” (Model 2). Except in the case where both doses were self-paying (“O–O”), children who received the first dose at their own expense (“O-N”, “O-F”) always had lower VEs than children who received it free of charge (“F-N”, “F-O”, “F-F”), whether in the 1- or 2-dose subgroups. Details of VEs (95% CI) are shown in Table 2.

**Table 2:** Comparison differences in indicators of protective effect between subgroups in subcorhort and exclusion

Dataset	Models	Independent variables	subgroups		Indicators of protective effect		*P-*value
Subcohort	Cox proportional hazards model using IPW	Model 1: Gender and Dose	0-dose vs	1-dose	VEs (95% CI)	82.54% (78.88%, 85.57%)	< 0.05
				2-dose		97.91% (96.91%, 98.59%)	
		Model 2: Gender and Type	"N–N" vs	"O-N"		71.19% (63.54%, 77.17%)	< 0.05
				"F-N"		89.78% (86.36%, 92.33%)	
				"O-F"		97.68% (96.61%, 98.45%)	
				"F-O"		99.66% (99.06%, 99.88%)	
				"O–O"		99.60% (99.20%, 99.80%)	
				"F-F"		99.70% (99.57%, 99.79%)	
Exclusion	Sensitivity analysis based on logistic regression	Model 3: Age, Gender, Area, and Dose	1-dose vs	Intercept	ORs	−10.41	< 0.05
				2-dose		−2.43	
		Model 4: Age, Gender, Area, and Type	"O-N" vs	Intercept		−8.76	< 0.05
				"F-N"		−1.67	
				"F-F"		−2.51	
				"F-O"		−4.12	
				"O-F"		−16.50	0.973
				"O–O"		−16.11	0.975
		Model 5: Age, Gender, Area, and Reason	"L-N" vs	Intercept		−10.42	< 0.05
				"L–T"		−2.30	
				"E-N"		−13.86	0.993
				"L-I"		26.31	0.998
				"T-I"		26.94	0.997
				"L-L"		−14.89	0.971
				"T-L"		−1.21	0.231
				"T-E"		−14.15	0.975
				"E-L"		−14.91	0.999
				"E-T"		−14.43	0.993
				"E-E"		−13.97	0.997

*IPW* inverse probability weighting, *VE* vaccine effectiveness, *OR* odds ratio, *Dose* varicella vaccine dosage; Type: cost types for varicella vaccine (Note: N = None, O = One dose, F = Self-funded. For example, “O-N” indicates One dose self-funded, no subsequent vaccination. Other combinations are to be interpreted similarly.); Area: chhildren's residence in Changzhou districts and county; Reeson: variable of the excluded part depends on the validity of the vaccination (Note: N = None, E = Early dose, T = Timely dose, L = Late dose, and I = Invalid dose. For example, “L–T” indicates first dose late, and last dose timely. Other combinations are to be interpreted similarly.)

**Fig. 5:**
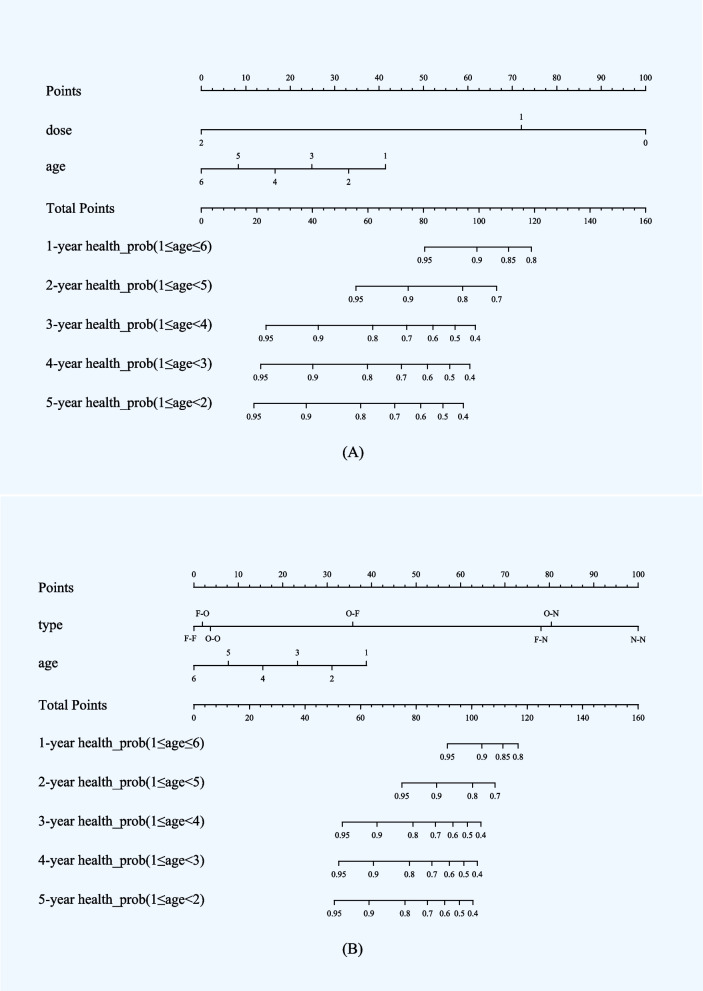
Nomograms predicted varicella by dose and cost type 1–5 years later. (The Nomograms quantify VEs by dose and cost type, and generate a cumulative score line, the Total Scores, which predict incidence over the next 1–5 years; (Note: N = None, O = One dose, F = Self-funded. For example, “O-N” indicates One dose self-funded, no subsequent vaccination. Other combinations are to be interpreted similarly.)

Figure 5 shows VEs as Nomograms. Age, doses, and cost types showed single-factor disease risks, while total points showed aggregate hazards, with larger points increasing risk. The Nomograms predicted disease-free survival after 1, 2, and 5 years based on total points. The 1-dose subgroup scored 72 points, while the 2-dose subgroup received 0. The Nomogram (Fig. 5A) quantified the 28% increase in protected points with 2 doses compared to 1 dose. The subgroups “F-O”, “O–O”, “O-F”, “F-N”, “O-N”, and “N–N” had points of 3, 5, 36, 78, 80, and 100, while “F-F” had 0. Personal age also affected total points. A 2-year-old who receives a single dose of a self-paying vaccine between 2016 and 2022 has a 90% chance of remaining varicella-free after 1 year, 75% after 2 years, and 40% after 3 years. The relevant data is in Fig. 5.

### Sensitivity analysis for the excluded dataset

We excluded 68,620 individuals from the original data. The characteristic variable distribution in this excluded subset is shown in Appendix 1. The results of Models 3 and 4 are consistent with the main study in Table 2. The significant results of Model 5 showed an intercept of −10.42, indicating that individuals had a minimal probability of developing varicella even with a single dose of vaccine and a delayed immunization. The estimated value of −2.30 for level “L–T” indicates that it was more effective than level “L-N,” which had only one delayed immunization dose (Table 2).

Appendix 2 shows a heat map of “Reason for exclusion” and “Types for cost”, demonstrating the excluded part’s characteristics. 1) Over 75% of the individuals excluded received a single dose of the vaccine late and free of charge; 2) More than 15% received the second dose on time and at their own expense after receiving the first dose late and free.

## Discussion

In the context of VarV not being included in the NIP, cities in China with the capacity to implement local immunization programs have evaluated VEs in the local high-coverage settings. As in other studies, this study found that two doses of varicella vaccine under the “1–4” vaccination program increased VEs sequentially. The study evaluated the timely benefits of implementing the vaccination program beyond just the VE after two doses.

### One-dose vs two-dose

Our study observed results consistent with vaccine effectiveness studies from multiple countries and regions [7, 20, 40, 41], indicating that a full two-dose vaccination strategy can establish a better immune barrier for the population. The following discussion expands on these findings, and provides a comprehensive view of the vaccine’s effectiveness. Studies summarizing the effectiveness of VarV in preventing varicella in the 25 years since the VarV vaccination program was implemented in the United States estimated that 1 dose of VarV provided moderately strong protection against the onset of varicella (71%−89%), and the pooled estimate of the VE of 2-dose VarV in preventing varicella was about 92%−95% [7, 40]. A meta-analysis of 32 observational studies published before 2018 on the VE of 1-dose and 2-dose VarV in Chinese children showed that the VEs were 75% (95% CI: 68% to 80%) and 90% (95% CI: 69% to 97%), respectively [41]. An observational study in Suzhou, a city in Jiangsu Province, evaluated the VEs of 1 and 2 doses of VarVs to be approximately 72.98% and 100%, respectively [20]. This city, also in Jiangsu Province, implemented a similar VarV vaccination program earlier than Changzhou.

The decline in varicella incidence probability with age (Fig. 5) was attributed to increased vaccine coverage, not age as a protective factor. This increase in coverage has resulted in a stronger population immune barrier among the vaccinated age group (1–6 years), which is relatively smaller compared to the at-risk population of varicella (2–14 years). Our findings indicate that higher coverage, rather than age, was key in reducing incidence. However, the long-term vaccine effectiveness under high coverage remained unclear, necessitating further study [16, 22, 42, 43]. This does not imply primary vaccine failure, but rather partial protection or inadequate induction of a fully protective immune response, as both hospitalization and severe disease rates were also reduced in children who became ill after VarV vaccination [7, 15]. A study analyzing the epidemiologic characteristics of varicella in Germany from 2009 to 2017 found that the incidence of the disease decreased with age in the age group under 9 years, with decreases in the number of cases ranging from fourfold to more than 20-fold [42]. While our study underscores the benefits of increased coverage, the potential efficacy decline over time, as discussed in other studies [16, 22, 42, 43], underscores the need for continued monitoring to assess long-term vaccine performance.

### High coverage, stronger immune barrier

In order to analyze the policy benefits of the implementation of the vaccination program, we divided seven subgroups using the variable “Type” for the type of vaccine cost, details shown in the part “ Methods”. Since VarVs were charged on their own before the vaccination program, if people’s type contains the character “F”, it means that they have benefited from the policy. However, given the timing of the vaccination, the “O–O” type is an exception. Even during the implementation of the vaccination program, a certain number of high-income families will opt for self-paying imported vaccines. For those children born before January 1, 2019, receiving a single dose of the vaccine appears to put them at higher risk of varicella than not receiving VarV at all. From January 1, 2019, this “paradox” seems to have disappeared. The survival analysis curves provide additional information in Figs. 2, 3, and 4.

On the one hand, when VarV was not included in the vaccination program, varicella had a higher risk of transmission in the susceptible population (children aged 2–14 years) during this period (born before January 1, 2019). Children willing to pay out-of-pocket for 1 dose of vaccine are likely to do so because they are perceived to be at high risk (excluding emergency doses due to varicella outbreaks). Their underlying risk of disease is higher than that of other children, and this risk cannot be effectively addressed by the “personal act” of self-paying vaccination. On the other hand, children born before January 1, 2019, who received the free 1-dose VarV were not “timely”, and the wide confidence intervals implied that “delayed vaccination” would reduce the VE. When the vaccination program is implemented, single-dose VarV coverage increases to > 95%, and the first dose receipt is earlier, effectively reducing the number of susceptible individuals. Children born during this period have a lower background risk of varicella than older children.

The study of varicella vaccination strategies, coverage, and varicella incidence in the United States also suggests that maintaining high 2-dose vaccination coverage has a significant impact on varicella epidemiologic trends [40]. In a study of the epidemiology of varicella outbreaks in Gansu, China, 28.4% of cases were found to have received 1-dose VarV, and only a single dose of self-pay VarV was recommended for children over 1 year of age in that province [44]. Lianyungang City, Jiangsu Province, studied the long-term VEs with 1 dose at intermediate levels of coverage in the context of no free vaccination program. The adjusted VE for 1 dose decreased from 72.9% to 41.8% [22]. From a public health perspective, a 2-dose complete vaccination program is more protective than a self-paying vaccination because high vaccination coverage strengthens the herd immunity barrier and effectively prevents breakthrough infections. In order to reduce the incidence of varicella, the Jiangsu Provincial Government has decided to incorporate varicella vaccination into the routine childhood immunization program from January 1, 2023, which is basically the same as that of Changzhou [22].

#### Timely vaccination for better protection

Sensitivity analysis results are robust to the primary results of this study. Results based on the logistic regression model suggest that two doses of vaccine with a timely second dose may be more effective in preventing varicella than a single dose of delayed vaccine (Table 2). Heat map (Appendix 2) based on the variables “Types for cost” and “Reasons for exclusion”: The exclusion of more than almost seventy thousand “delayed” individuals likely resulted in an overestimation of the VE of the first-dose vaccine and an underestimation of the VE of the self-paying VarVs. These findings are an indication of the importance of timely vaccination.

This study has the following limitations. 1). During the 2019 coronavirus disease (COVID-19) pandemic, China implemented a strict social distance control policy for prevention and control, resulting in a significant reduction in the incidence of all respiratory infections during this period. A VE study in Shanghai found a significant reduction in varicella cases in 2020, 54% of which was attributed to social distancing for COVID-19 [45]. 2). And then, the incidence of varicella may also have been underestimated because of the passive surveillance system with a rate of missed reports and differences in people’s willingness to seek medical care (those who pay for their own vaccinations may be more concerned about their own health status and actively seek medical care). However, this study maintained a high level of representativeness for the included varicella cases due to the infectious disease information system’s strict training, management, and operational systems. 3). Our study assumed constant VZV transmission, potentially overlooking transmission variability. This simplification may have affected the precision of our vaccine effectiveness estimates. A more dynamic model in future research could enhance our understanding of VZV epidemiology. 4). Another limitation of our study was the use of 14 days to determine the lag time for the vaccine’s effectiveness, which failed to encompass the 42-day maximum incubation period for varicella breakthrough cases [46]. This could have resulted in an underestimation of the vaccine’s protective effect. Therefore, this study may overestimate the short-term VEs of 2-dose VarVs in three years, and a long-term follow-up observation is necessary in the future.

Although the direct extrapolation of our results to the entire Jiangsu Province or China is not feasible, the robust methodology employed in our study holds significant potential for application in other regions or countries equipped with comparable surveillance systems, thereby expanding the global significance of our research findings.

## Conclusion

In conclusion, the “1–4” Vaccination Program in Changzhou, which has been in place for three years, is effectively improving coverage and timeliness of vaccination. Improving the 2-dose VarV vaccination coverage has a significant impact on the immune barrier. The timely benefits of vaccination for susceptible children were significant.

## Figures and Tables

**Table:**
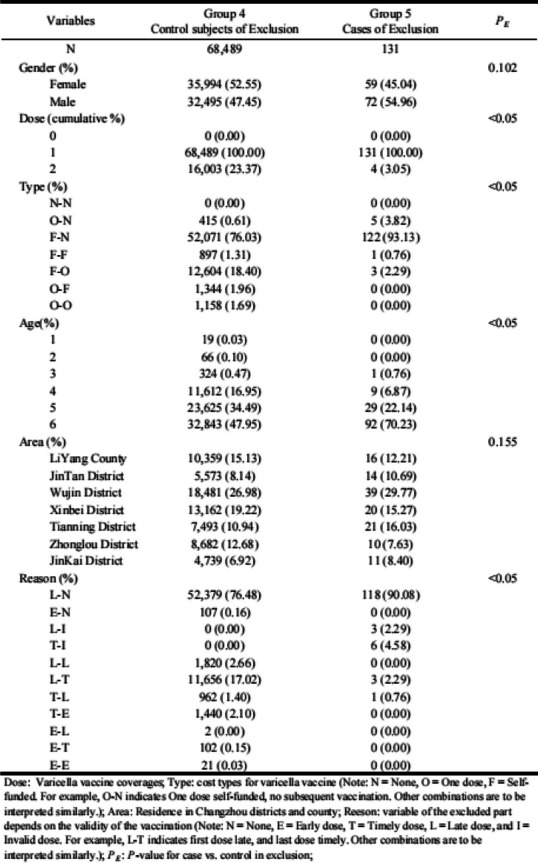
Characteristics of the exclusion

**Figure:**
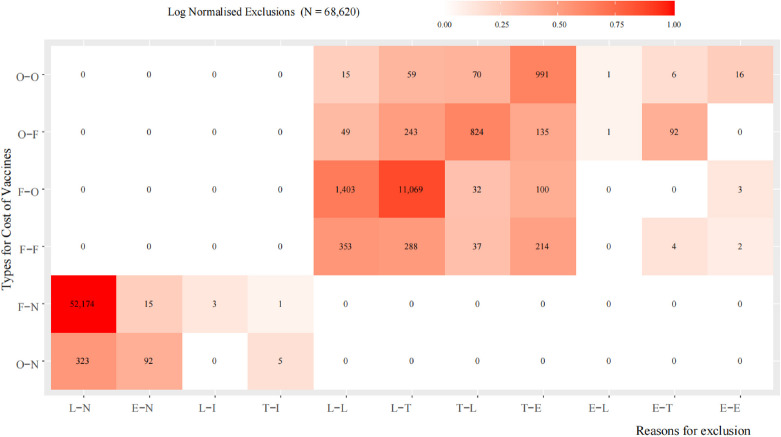
A heat map of reasons for exclusion and types for cost shows the characteristics of the excluded area. Logarithmic transformation normalized graph data to increase heat map resolution. (Note: N = None, O = One dose, F = Self-funded. For example, “O-N” indicates One dose self-funded, no subsequent vaccination. Other combinations are to be interpreted similarly. Note: N = None, E = Early dose, T = Timely dose, L = Late dose, and I = Invalid dose. For example, “L-T” indicates the first dose late, and the last dose timely. Other combinations are to be interpreted similarly)

## Data Availability

The study data are available for academic purposes upon reasonable request. To gain access, please contact the primary corresponding author Xufeng Lv for further details.

## Abbreviations

VZV: Varicella-zoster virus
WHO: World Health Organization
VarV: Varicella vaccine
NIP: National Immunization Program
NNIDSS: National Notifiable Infectious Disease Surveillance System
VE: Vaccine effectiveness
CCS: Case Cohort Study
HR: Hazard ratio
CI: Confidence interval
IIS: Immunization information system
IPW: Inverse probability weighting
COVID-19: 2019 Coronavirus diseases
